# Trends in lumbar epidural injection selection: A survey of practitioner preferences and practice patterns

**DOI:** 10.1016/j.inpm.2025.100607

**Published:** 2025-06-29

**Authors:** Ryan Triglia, Andrew Walrond, Jesse Wagner, Paul M. Kitei, Jeffrey Boyd, Jeremy I. Simon

**Affiliations:** a Thomas Jefferson University Hospital, Philadelphia, Pennsylvania, USA; b Rothman Orthopaedic Institute, Philadelphia, Pennsylvania, USA; c Pennsylvania Pain & Spine Institute, Chalfont, Pennsylvania, USA

## Abstract

**Background:**

Lumbar radiculopathy is estimated to affect approximately 3–5 % of the population. Among the leading causes of radiculopathy are degenerative or congenital spinal stenosis and lumbar disc herniations, which can contribute to compression and narrowing in various regions of the spine including the neural foramen, subarticular recess, or central canal. When patients do not respond to typical conservative treatment such as medications and physical therapy, epidural steroid injections can be considered as a next step in management. There are three approaches available for accessing the lumbar epidural space: caudal, interlaminar, and transforaminal. There is no clear consensus regarding the selected approach for an epidural injection based on a patient's history, physical examination, and imaging findings, however.

**Objective:**

The purpose of this study was to explore how factors such as primary residency training, fellowship training, practice setting, adherence to IPSIS guidelines, geographic location, and years of experience may influence epidural approach preferences.

**Methods:**

A survey was created utilizing the Survey Monkey™ platform which was then administered by the International Pain and Spine Intervention Society (IPSIS) to all active members via email. The survey consisted of seven questions asking for demographic information including residency specialty, fellowship training, if the fellowship emphasized IPSIS guidelines, years in practice, country of practice, and practice setting. There were questions that described hypothetical clinical scenarios that provided the respondent with the pain distribution and the associated pertinent magnetic resonance imaging (MRI) findings. For each scenario, the responder was given options for type of therapeutic injection the practitioner would choose. The final two questions then asked which steroid the responder would utilize for an interlaminar and transforaminal epidural steroid injection. The survey was open for completion during a three-month period. A total of 202 IPSIS members responded, with an average of 196 responses to each question with a completion rate of 74 %.

**Results:**

Most respondents completed residency in Physical Medicine and Rehabilitation (63.1 %) or Anesthesiology (29.9 %), with 67.7 % reporting fellowship training aligned with IPSIS guidelines. The most common fellowship type was ACGME-accredited pain (38.6 %), and respondents were primarily based in the U.S. (84.8 %), practicing in private multi-specialty groups (35.5 %). Experience levels were well distributed, with most in either early (<5 years, 32.1 %) or late-career (>15 years, 34.7 %) stages. Across all six clinical scenarios, the transforaminal supraneural approach was most frequently selected, especially at L4-L5 and L5-S1. Respondents selecting the most common techniques were primarily PM&R-trained and fellowship-trained in ACGME pain programs using IPSIS-guided approaches, with a balanced distribution across years in practice. Dexamethasone was the most frequently used steroid for both interlaminar (35.1 %) and transforaminal (71.1 %) epidural injections. Providers selecting dexamethasone were again predominantly PM&R-trained, IPSIS-guided, and either early or late career.

**Conclusion:**

Symptom distribution, particularly radicular pain, strongly influenced the choice of transforaminal supraneural injections, underscoring the primacy of clinical presentation over imaging alone. The predominant use of dexamethasone reflects a broader shift toward safety-oriented protocols. Overall, provider training and practice setting were more predictive of decision-making than clinical scenario complexity, highlighting the need for continued education across all provider types.

## 1 Introduction

Lumbar radiculopathy is estimated to affect approximately 3–5 % of the population [1]. Among the leading causes of radiculopathy are lumbar disc herniations and lumbar spondylosis, which can contribute to neural compression in various regions such as the foramen, subarticular recess, or central canal. Additionally, patients with lumbar radiculopathy may present with synovial cysts that are situated near or compressing nerves within the spinal canal or foramen. The incidence of painful lumbar disc herniations is estimated to range from 5 to 20 cases per 1000 adults annually [2]. Symptomatic lumbar spinal stenosis is estimated to affect anywhere between 19 and 47 % of individuals in the United States aged 60 or older [3].

Lumbar radiculopathy has the potential to resolve spontaneously or with minimal medical intervention. Symptomatic disc herniations often undergo spontaneous resorption without requiring surgical intervention. A study by Kesikburun et al. demonstrated that 75 % of lumbar disc extrusions showed complete resolution on MRI at 17 months following the initial evaluation [4]. Conservative treatment approaches typically involve physical therapy, relative rest and avoidance of aggravating factors. Patients may also find relief through over-the-counter or prescription analgesics, such as non-steroidal anti-inflammatory medications (NSAIDs), muscle relaxants, gabapentinoids, and opioids. However, it is important to acknowledge that opioid prescribing practices have shifted significantly in response to the ongoing opioid crisis and updated CDC guidelines, resulting in more cautious and limited use, generally reserved for cases of severe and intractable pain.

In cases where patients do not respond to the aforementioned treatments, epidural steroid injections (ESIs) may be considered. The rationale is to administer corticosteroid directly to the affected nerve root area to reduce inflammation. There are three approaches for accessing the lumbar epidural space: caudal, interlaminar, and transforaminal. The caudal approach involves needle insertion into the sacral canal via the sacral hiatus, avoiding advancement beyond the S3 level to prevent dural puncture. The lumbar interlaminar approach (L-ILESI) involves inserting a needle between adjacent laminae, either centrally or para-centrally, using “loss of resistance” to pressure on a syringe, and confirming with contrast injection by obtaining a lateral and/or contralateral oblique image [4]. The lumbar transforaminal epidural steroid injection (L-TFESI) targets the epidural space through the foramen through which the spinal nerve exits, delivering medication over or beneath the nerve (supraneural or infraneural technique). Due to the risk of inadvertent arteriole cannulation, the use of particulate steroids in L-TFESIs is generally discouraged [[6], [7], [8]].

Although transforaminal injections are often regarded as more “selective,” studies, such as those by Furman et al. suggest that injectate may still spread beyond the targeted level [9]. Moreover, evidence remains conflicting regarding the superiority of one approach over another for treating lumbar radiculopathy [10]. Interventional spine practitioners frequently face challenges in selecting the most appropriate epidural technique, often relying on a combination of clinical history, examination findings, and imaging.

While there are established contraindications to certain techniques—such as avoiding interlaminar injections at surgically altered levels where the ligamentum flavum is compromised-the epidural approach is often made subjectively. Informal discussions among colleagues often reveal different choices shaped by personal training or experience, procedural outcomes, and institutional preferences. Prior studies have sought to elucidate procedural preferences and practice patterns among interventionalists, though findings have varied [6]. This study attempted to identify trends in lumbar epidural approach among practitioners given different clinical scenarios. It also examined whether factors such as primary residency training, fellowship background, clinical practice setting, adherence to IPSIS guidelines during training, geographic location, and years of clinical experience influence procedural choices.

## 2 Methods

### 2.1 Survey design and dissemination

An anonymous English-language survey consisting of 15 questions was developed and distributed via SurveyMonkey™ to assess physician preferences and procedural selection in the context of lumbar epidural steroid injections. The survey was adapted and approved by members of the IPSIS Research Committee and was modeled after a similar study evaluating practice patterns for genicular nerve radiofrequency ablation [13]. The questionnaire included seven items capturing demographic information including primary residency specialty, fellowship training, emphasis on IPSIS guidelines during training, years in practice, country of practice, and if within the United States, state of practice. Respondents were asked their current practice setting (e.g., academic, private group, hospital-employed). Six questions described hypothetical clinical scenarios involving lumbar radiculopathy, including pain complaints and MRI findings, with respondents asked their preferred therapeutic epidural approach. The final two questions assessed preferences regarding steroid type for interlaminar and transforaminal injections.

The survey read as follows:

1. What was your residency training?
  a. Anesthesia
  b. Physical Medicine and Rehabilitation
  c. Neurology
  d. Psychiatry
  e. Radiology
  f. Other (please specify)
2. What type of fellowship did you complete?
  a. ACGME Pain
  b. ACGME Sports
  c. NASS ISMM (Interventional Spine)
  d. Non-accredited spine
  e. Non-accredited sports (or sports and spine)
  f Other (please specify)
3. Did your interventional training emphasize the IPSIS guidelines-based approach to spinal injections?
  a. Yes
  b. No
  c. Other (please specify)
4. How many years have you been in practice?
  a. Less than 5 years
  b. 5–10 years
  c. 10–15 years
  d. Greater than 15 years
5. In which country do you practice?
  a. United States of America
  b. Canada
  c. United Kingdom
  d. Australia
  e. Other (please specify)
6. Which state within the United States do you practice in? If outside of the United States, which province?
7. Which of the following best describes your current practice environment?
  a. Solo practitioner
  b. Group practice, single specialty, private practice
  c. Multi-specialty group, private practice
  d. Hospital employed
  e. Academic institution
  f. Other (please specify

Answer choices for questions 8-15.

A. Right L5-S1 interlaminar
B. Right L4-5 interlaminar
C. Right L4-5 transforaminal infraneural approach
D. Right L4-5 transforaminal supraneural approach
E. Right L5-S1 transforaminal infraneural approach
F. Right L5-S1 transforaminal supraneural approach
G. Right L4-5 and L5-S1 transforaminal infraneural approach
H. Right L4-5 and L5-S1 transforaminal supraneural approach
I. Caudal
J. Would not perform

#### 2.1.1 Scenario 1

An otherwise healthy 55-year-old male presents with RIGHT LOWER BACK EQUAL TO RIGHT LEG PAIN. You have elected to perform an epidural steroid injection. MRI of the lumbar spine demonstrates a moderate sized right L4-5 paracentral disc herniation compressing the traversing L5 nerve root with mild subarticular stenosis. All routes of access appear accessible on imaging.

Question 8: Which therapeutic epidural procedure would you perform?

#### 2.1.2 Scenario 2

An otherwise healthy 55-year-old male presents with RIGHT LOWER BACK EQUAL TO RIGHT LEG PAIN. You have elected to perform an epidural steroid injection. MRI of the lumbar spine demonstrates moderate neural foraminal stenosis at L4-5. All routes of access appear accessible on imaging.

Question 9: Which therapeutic epidural procedure would you perform?

#### 2.1.3 Scenario 3

An otherwise healthy 55-year-old male presents with RIGHT LOWER BACK EQUAL TO RIGHT LEG PAIN. You have elected to perform an epidural steroid injection. MRI of the lumbar spine demonstrates moderate spinal canal stenosis at L4-5. All routes of access appear accessible on imaging.

Question 10: Which therapeutic epidural procedure would you perform?

#### 2.1.4 Scenario 4

An otherwise healthy 55-year-old male presents with RIGHT LEG PAIN WITH MINIMAL RIGHT LOW BACK PAIN. You have elected to perform an epidural steroid injection. MRI of the lumbar spine demonstrates a moderate sized right L4-5 paracentral disc herniation compressing the traversing L5 nerve root with mild subarticular stenosis. All routes of access appear accessible on imaging.

Question 11: Which therapeutic epidural procedure would you perform?

#### 2.1.5 Scenario 5

An otherwise healthy 55-year-old male presents with RIGHT LEG PAIN WITH MINIMAL RIGHT LOWER BACK PAIN. You have elected to perform an epidural steroid injection. MRI of the lumbar spine demonstrates moderate neural foraminal stenosis at L4-5. All routes of access appear accessible on imaging.

Question 12: Which therapeutic epidural procedure would you perform?

#### 2.1.6 Scenario 6

An otherwise healthy 55-year-old male presents with RIGHT LEG PAIN WITH MINIMAL RIGHT LOWER BACK PAIN. You have elected to perform an epidural steroid injection. He has MRI of the lumbar spine demonstrates moderate spinal canal stenosis at L4-5. All routes of access appear accessible on imaging.

Question 13: Which therapeutic epidural procedure would you perform?

Answer choices for questions 14 and 15.

A. Methylprednisolone
B. Triamcinolone
C. Betamethasone
D. Dexamethasone

Question 14: Which steroid would you choose for an interlaminar epidural injection?

Question 15: Which steroid would you choose for a transforaminal epidural injection?

### 2.2 Institutional review board (IRB) status

This study was deemed exempt by Thomas Jefferson University Institutional Review Board (IRB) as it involved anonymous survey data collection without any intervention or interaction with human subjects.

### 2.3 Data collection

The data was stored on Survey Monkey™ The survey was administered through electronic mail to members of IPSIS from November 2023 to January 2024. A total of 202 responses were obtained. The authors filtered the results by the responses to the first 7 questions and applied them to each clinical scenario as well as the final 2 questions regarding steroids. Responses that less than 10 % were deemed clinically insignificant and not reported in results. The completion rate was 74 % with an average completion time of 6 min.

## 3 Results

### 3.1 Survey respondent characteristics

The majority of respondents reported completing their residency training in Physical Medicine and Rehabilitation (PM&R) (63.08 %), followed by Anesthesiology (29.90 %) as shown below in Table 1, with other specialties making up a small minority. Over two-thirds (67.69 %) indicated their fellowship training emphasized an IPSIS guideline-based approach. The most common fellowship type was ACGME-accredited pain (38.58 %), with non-accredited spine and NASS ISMM (Interventional Spine) fellowships following at 15.74 % and 14.21 %, respectively. Notably, 12.18 % reported having no fellowship training at all. In terms of experience, the distribution was fairly even, though a slight majority had either more than 15 years (34.69 %) or less than 5 years (32.14 %) in practice, suggesting both seasoned and early-career practitioners were well represented. Geographically, the vast majority (84.75 %) practiced in the United States, with other countries each contributing a very small fraction of responses. Practice settings varied, with the most common being multi-specialty private group practices (35.53 %), followed by hospital-employed roles (21.83 %) and single-specialty private group practices (21.33 %). A smaller proportion worked in academic institutions (10.66 %) or as solo practitioners (7.61 %). Overall, the data suggest that respondents are predominantly U.S.-based, trained in PM&R or Anesthesiology, and work in group private practices, often with specialized pain fellowship backgrounds aligned with IPSIS guidelines.

**Table 1:** Survey respondent characteristics.

		Frequency	Percentage
**Residency**	Physical Medicine and Rehabilitation	123	63.08 %
	Anesthesiology	58	29.90 %
	Other	10	5.12 %
	Neurology	2	1.03 %
	Radiology	2	1.03 %
**IPSIS Adherence**	Yes	132	67.69 %
	No	58	29.90 %
	Other	7	3.61 %
**Fellowship**	ACGME Pain	76	38.58 %
	Non-accredited Spine	31	15.74 %
	NASS ISMM (Interventional Spine)	28	14.21 %
	None	24	12.18 %
	Non-accredited Sports (or Sports and Spine)	18	9.14 %
	Other	15	7.61 %
	ACGME Sports	5	2.54 %
**Years in Practice**	Greater than 15 years	68	34.69 %
	10–15 years	29	14.80 %
	5–10 years	36	18.37 %
	Less than 5 years	63	32.14 %
**Country of Practice**	United States of America	167	84.75 %
	United Kingdom	6	3.05 %
	Canada	5	2.54 %
	Ireland	3	1.52 %
	New Zealand	2	1.02 %
	Germany	2	1.02 %
	Other	10	5.10 %
**Current Practice Environment**	Multi-specialty group, private practice	70	35.53 %
	Hospital employed	43	21.83 %
	Group practice, single specialty, private practice	42	21.33 %
	Academic institution	21	10.66 %
	Solo practitioner, private practice	15	7.61 %
	Other (please specify)	6	3.05 %

### 3.2 Epidural injection approach

Analysis of the six clinical scenarios demonstrates consistent trends in the selection of therapeutic epidural injection techniques based on symptom distribution and MRI findings. Across all cases, the transforaminal supraneural approach, particularly at L4-L5 or L5-S1, was most commonly chosen (as seen in Table 2). In Scenario 1, which described right lower back pain equal to right leg pain with a right L4-5 paracentral disc herniation compressing the L5 nerve root, the right L5-S1 transforaminal supraneural approach was most selected (n = 66, 34.0 %). Similarly, Scenario 2, presenting the same symptoms but with L4-5 foraminal stenosis, saw the right L4-5 transforaminal supraneural approach as the predominant choice (n = 97, 50.0 %). In Scenario 4, where right leg pain exceeded back pain with a similar herniation at L4-5, the right L5-S1 transforaminal supraneural approach was again the most selected (n = 69, 35.60 %). This trend continued in Scenario 5, featuring right leg pain greater than back pain and L4-5 foraminal stenosis, where the right L4-5 transforaminal supraneural approach led with (n = 88, 45.40 %). Notably Scenario 3, which involved moderate spinal canal stenosis at L4-5 with symptoms equal in the back and leg, saw a shift—though modest—toward the interlaminar approach (n = 38, 19.60 %), indicating that central canal stenosis may slightly influence route selection. However, in Scenario 6, despite central canal stenosis, the predominance of leg pain over back pain led respondents back to favor the right L5-S1 transforaminal supraneural approach (n = 35, 18.0 %).

**Table 2:** Epidural injection approach by scenario.

Scenario	Injection Approach	Frequency (%)
**1**	Right L5-S1 transforaminal supraneural	66 (34.0 %)
	Right L4-5 interlaminar	27 (13.9 %)
	Right L4-5 and L5-S1 transforaminal supraneural	26 (13.4 %)
	Right L5-S1 interlaminar	18 (9.28 %)
	Right L4-5 transforaminal infraneural	13 (6.70 %)
	Right L4-5 transforaminal supraneural	12 (6.19 %)
	Right L4-5 and L5-S1 transforaminal infraneural	8 (4.12 %)
	Other	5 (2.58 %)
	Right L5-S1 transforaminal infraneural	4 (2.06 %)
	Caudal	3 (1.55 %)
**2**	Right L4-5 transforaminal supraneural	97 (50.0 %)
	Right L4-5 interlaminar	27 (13.9 %)
	Right L4-5 transforaminal infraneural	20 (10.3 %)
	Right L4-5 and L5-S1 transforaminal supraneural	9 (4.64 %)
	Right L4-5 and L5-S1 transforaminal infraneural	5 (2.58 %)
	Other	4 (2.06 %)
	Caudal	3 (1.55 %)
	Right L5-S1 interlaminar	3 (1.55 %)
	Right L5-S1 transforaminal infraneural	2 (1.03 %)
	Right L5-S1 transforaminal supraneural	2 (1.03 %)
**3**	Right L4-5 interlaminar	38 (19.6 %)
	Right L5-S1 transforaminal supraneural	37 (19.1 %)
	Right L5-S1 interlaminar	33 (17.0 %)
	Right L4-5 and L5-S1 transforaminal supraneural	18 (9.28 %)
	Right L4-5 transforaminal supraneural	17 (8.76 %)
	Caudal	8 (4.12 %)
	Right L4-5 transforaminal infraneural	9 (4.64 %)
	Other	5 (2.58 %)
	Right L4-5 and L5-S1 transforaminal infraneural	2 (1.03 %)
	Right L5-S1 transforaminal infraneural	1 (0.52 %)
**4**	Right L5-S1 transforaminal supraneural	69 (35.6 %)
	Right L4-5 and L5-S1 transforaminal supraneural	25 (12.9 %)
	Right L4-5 interlaminar	14 (7.22 %)
	Right L4-5 transforaminal supraneural	13 (6.70 %)
	Right L4-5 and L5-S1 transforaminal infraneural	9 (4.64 %)
	Right L4-5 transforaminal infraneural	8 (4.12 %)
	Right L5-S1 interlaminar	8 (4.12 %)
	Right L5-S1 transforaminal infraneural	8 (4.12 %)
	Other	4 (2.06 %)
	Caudal	3 (1.55 %)
**5**	Right L4-5 transforaminal supraneural	88 (45.4 %)
	Right L4-5 transforaminal infraneural	22 (11.3 %)
	Right L4-5 and L5-S1 transforaminal supraneural	13 (6.70 %)
	Right L4-5 interlaminar	13 (6.70 %)
	Right L5-S1 transforaminal supraneural	6 (3.09 %)
	Other	4 (2.06 %)
	Caudal	3 (1.55 %)
	Right L4-5 and L5-S1 transforaminal infraneural	3 (1.55 %)
	Right L5-S1 interlaminar	2 (1.03 %)
	Would not perform	1 (0.52 %)
**6**	Right L5-S1 transforaminal supraneural	35 (18.0 %)
	Right L4-5 interlaminar	30 (15.5 %)
	Right L5-S1 interlaminar	26 (13.4 %)
	Right L4-5 and L5-S1 transforaminal supraneural	21 (10.8 %)
	Right L4-5 transforaminal supraneural	14 (7.22 %)
	Right L4-5 transforaminal infraneural	8 (4.12 %)
	Caudal	5 (2.58 %)
	Other	5 (2.58 %)
	Right L4-5 and L5-S1 transforaminal infraneural	5 (2.58 %)
	Right L5-S1 transforaminal infraneural	2 (1.03 %)

### 3.3 Respondent training and practice

Across all scenarios, respondents who selected the most common answer completed Physical Medicine and Rehabilitation residencies (e.g., Scenario 2: n = 78, 78.59 %) and ACGME-accredited pain fellowships (Scenario 5: n = 29, 32.22 %), with many trained under the IPSIS guidelines for spinal injection practices (as seen in Table 3). Respondents were split in years of experience, with notable representation among both early-career (e.g., Scenario 3: n = 16, 41.03 % practiced <5 years) and late-career physicians (e.g., Scenario 6: n = 17, 47.22 % practiced >15 years).

**Table 3:** Respondent characteristics of most commonly selected answer of scenarios 1–6.

	Scenario 1	Scenario 2	Scenario 3	Scenario 4	Scenario 5	Scenario 6
	Right L5-S1 TF Supraneural (67, 34.01 %)	Right L4-L5 TF Supraneural (99, 50.25 %)	Right L4-L5 Interlaminar (39, 19.80 %)	Right L5-S1 TF Supraneural (70, 35.53 %)	Right L4-L5 TF Supraneural (90, 45.69 %)	Right L5-S1 TF Supraneural (37, 18.78 %)
**Residency**						
PM&R	54 (80.60 %)	78 (78.59 %)	22 (57.89 %)	54 (77.14 %)	72 (80.90 %)	34 (91.89 %)
Anesthesia	10 (14.93 %)	19 (19.39 %)	13 (34.21 %)	12 (17.14 %)	14 (15.73 %)	2 (5.41 %)

**Fellowship**						
Pain	15 (22.39 %)	29 (29.29 %)	18 (46.15 %)	22 (31.43 %)	29 (32.22 %)	4 (10.81 %)
Non-Acc Spine	14 (20.90 %)	17 (17.17 %)	–	13 (18.57 %)	17 (18.89 %)	11 (29.73 %)
NASS ISMM	11 (16.42 %)	21 (21.21 %)	5 (12.82 %)	10 (14.29 %)	18 (20.00 %)	5 (13.51 %)
Non-Acc Sports	7 (10.45 %)	10 (10.10 %)	4 (10.26 %)	–	10 (11.11 %)	4 (10.81 %)
None	12 (17.91 %)	15 (15.15 %)	6 (15.38 %)	12 (17.14 %)	10 (11.11 %)	8 (21.62 %)

**IPSIS Emphasis**						
Yes	45 (67.16 %)	73 (73.74 %)	26 (66.67 %)	49 (70.00 %)	72 (80.00 %)	27 (72.97 %)
No	20 (29.85 %)	23 (23.23 %)	13 (33.33 %)	20 (28.57 %)	16 (17.78 %)	8 (21.62 %)

**Years in Practice**						
<5	15 (22.73 %)	35 (35.71 %)	16 (41.03 %)	20 (28.99 %)	26 (29.21 %)	6 (16.67 %)
5–10	13 (19.70 %)	17 (17.35 %)	8 (20.51 %)	15 (21.74 %)	20 (22.47 %)	8 (22.22 %)
10–15	14 (21.21 %)	16 (16.33 %)	–	10 (14.49 %)	18 (20.22 %)	5 (13.89 %)
>15	24 (36.36 %)	30 (30.61 %)	13 (33.33 %)	24 (34.78 %)	25 (28.09 %)	17 (47.22 %)

**Practice**						
Multi Specialty	25 (37.31 %)	38 (38.38 %)	15 (38.46 %)	24 (34.29 %)	36 (40.00 %)	12 (32.43 %)
Single Specialty	12 (17.91 %)	21 (21.21 %)	10 (25.64 %)	14 (20.00 %)	17 (18.89 %)	6 (16.22 %)
Hospital	14 (20.90 %)	20 (20.20 %)	8 (20.51 %)	17 (24.29 %)	20 (22.22 %)	7 (18.92 %)
Academic	12 (17.91 %)	14 (14.14 %)	5 (12.82 %)	12 (17.14 %)	12 (13.33 %)	9 (24.32 %)

### 3.4 Injectate preference

The most commonly selected injectate choice for an interlaminar epidural injection was dexamethasone (n = 68, 35.10 %) (Table 4). The majority graduated from a physical medicine and rehabilitation residency (n = 47, 69.12 %) and completed ACGME pain fellowship (n = 24, 35.29 %) emphasizing the IPSIS guidelines-based approach to spinal injections. Most respondents practiced for greater than 15 years (n = 26, 38.24 %) or less than 5 years (n = 21, 30.88 %) (see Table 5).

**Table 4:** Injectate choice for an ILESI.

Steroid	Frequency	Percentage
Dexamethasone	68	**35.1 %**
Methylprednisolone	32	**16.5 %**
Triamcinolone	32	**16.5 %**
Betamethasone	16	**8.25 %**
Other	3	**1.55 %**

**Table 5:** Characteristics of respondent for most commonly selected injectate for ILESI.

		Frequency	Percentage
Most commonly selected medication	Dexamethasone	68	34.52 %
Residency Completed	Physical Medicine and Rehabilitation Residency	47	69.12 %
	Anesthesia Residency	16	23.53 %
Fellowship Completed	ACGME Pain Fellowship	24	35.29 %
	Non-accredited Spine Fellowship	12	17.65 %
	None	11	16.18 %
	Non-accredited Sports (or Sports and Spine) Fellowship	7	10.29 %
Emphasis on IPSIS Guidelines in Training	Yes	48	70.59 %
	No	18	26.47 %
Years in Practice	Greater than 15 years	26	38.24 %
	Less than 5 years	21	30.88 %
	10–15 years	11	16.18 %
	5–10 years	10	14.71 %

The most commonly selected injectate for a transforaminal epidural injection was dexamethasone (n = 140, 71.07 %) (Table 5). The majority graduated from a physical medicine and rehabilitation residency (n = 92, 66.19 %) and completed a ACGME pain fellowship (n = 50, 35.71 %) emphasizing the IPSIS guidelines-based approach to spinal injections. Many practitioners have been practicing greater than 15 years (n = 52, 37.41 %), and less than 5 years (n = 44, 31.65 %).

## 4 Discussion

### 4.1 Epidural injection approach

Across the six clinical scenarios, the transforaminal supraneural approach was the most consistently chosen technique, particularly at L4–L5 and L5–S1 levels in cases involving radicular pain from foraminal stenosis or paracentral disc herniation. This preference demonstrates a clear clinical emphasis on targeted delivery of corticosteroids to the affected nerve root, especially in cases where radicular symptoms predominate. The predominance of this approach aligns with literature citing superior efficacy and drug delivery precision for transforaminal over interlaminar or caudal techniques in managing lumbar radiculopathy [[14], [15], [16]]. In Scenarios 1, 2, 4, and 5, where imaging correlated with foraminal or paracentral disc pathology and symptoms favored a radicular pattern, more than 50 % of respondents selected the transforaminal supraneural route at the level correlating to the affected nerve.

Although there was a modest increase in interlaminar injection selection (19.6 %) for Scenario 3, the transforaminal route remained the predominant choice (43.3 %), suggesting that even in cases of diffuse central stenosis, many practitioners continue to favor transforaminal delivery, possibly due to its established efficacy and more targeted drug delivery. Prior research has shown that the interlaminar approach may better address diffuse epidural inflammation or multilevel stenosis, albeit with lower selectivity [17].

However, when central stenosis coexisted with predominantly radicular symptoms (as in Scenario 6), the preferred approach was transforaminal injections (right L5–S1 supraneural, n = 35, 18.0 %). This suggests that symptom pattern (radicular vs axial) plays a more influential role in approach selection than MRI findings alone, a finding echoed in recent literature supporting patient-centered, symptom-driven interventional decision-making [18].

### 4.2 Respondent training and practice

A trend across all scenarios is the influence of clinical training on decision-making. The majority of respondents who chose the most common (and guideline-concordant) injection techniques had completed PM&R residencies (over 70 % in most scenarios), ACGME-accredited pain fellowships, and training with an IPSIS emphasis.

These respondents demonstrated a higher rate of adherence to evidence-based approaches, particularly the selection of non-particulate steroids for transforaminal injections and the use of supraneural trajectories at appropriate levels. This supports previous findings that standardized pain fellowships and guideline exposure correlate with safer and more consistent interventional practices [19].

Years in practice also influenced technique preferences. Early-career physicians (0–5 years) frequently aligned with most commonly selected responses, suggesting a **stronger adherence to contemporary training paradigms.** Conversely, those in practice >15 years showed **slightly more variation,** possibly reflecting either evolving personal practice patterns or broader clinical judgment, potentially deviating from newer norms in nuanced or ambiguous cases [20].

Respondents in **academic and hospital-based environments** more often selected the most common answers, suggesting these clinicians are more likely to adhere to established standards and protocols. Potential reasons could include peer review pressure, academic oversight, or continuing education emphasis, but further studies would need to be done to confirm these possibilities. In contrast, **private practice respondents** showed greater variability, which may reflect increased autonomy or resource-driven constraints in procedural choices, but further study would be warrented. These patterns echo existing literature. Desai et al. (2010) found that practice patterns in epidural injections were significantly influenced by training background and institutional affiliation, with academic centers showing greater consistency and adherence to evidence-based approaches.

### 4.3 Injectate preference

Dexamethasone (non-particulate steroid) was shown to be the favored agent for both interlaminar (n = 68, 34.52 %) and transforaminal (n = 140, 71.07 %) injections. This aligns with current safety guidelines and large-scale reviews emphasizing the reduced risk of spinal cord infarction and embolic events with non-particulate agents via the transforaminal approach [21]. Of note, a significant number of respondents selected a particulate steroid, triamcinolone (n = 32, 16.50 %) and methylprednisolone (n = 32, 16.50 %) for interlaminar injectate. The variability in the choice of injectate for lumbar interlaminar epidural steroid injections was expected, as there are no reports of spinal cord infarction with particulate-containing medications with this approach. Interestingly, all triamcinolone preparations cite a contraindication for epidural or intrathecal use [9].

The majority of respondents chose dexamethasone as the therapeutic medication for lumbar transforaminal epidural steroid injections (L-TFESI) (as seen in Table 6). This is consistent with the guidelines as there are several case reports of spinal cord infarction with the use of particulate steroids in L-TFESIs [5,10,11]. There are also anatomic considerations that support this risk [12]. Interestingly, the second most commonly selected steroid for L-TFESIs was again triamcinolone. While only 5 respondents chose this answer (2.58 %), this is not consistent with the current guidelines and contrary to the black-box warning.

**Table 6:** Injectate choice for a TFESI.

Steroid	Frequency	Percentage
Dexamethasone	140	**71.07 %**
Triamcinolone	5	**2.58 %**
Betamethasone	4	**2.06 %**
Methylprednisolone	3	**1.55 %**
Other (please specify)	2	**1.03 %**

While there are studies showing non-inferiority of dexamethasone compared to other steroids [13], there are practitioners choosing to use particulate preparations for L-TFESIs even within IPSIS.

Other investigators have looked at practice patterns amongst interventionalists. A study by Rathmell et al. looked at consensus opinions in preventing potential complications in the performance of epidural steroid injections. This expert collaboration concluded that 17 factors would improve safety with LTFESIs and ILESIs [14]. Gureck et al. surveyed accredited pain fellowships directors regarding TFESIs and found significant variability in safety practices [7]. Bingham et al. surveyed 261 interventionalists regarding adherence to 2015 multidisciplinary guidelines. This study also showed variability in practice patterns and observance of expert recommendations [8].

### 4.4 Study limitations and future considerations

This study has several limitations. First, there was a disproportionate representation of PM&R physicians among IPSIS respondents, which may have skewed the results toward PM&R-focused practice patterns. Second, the clinical scenarios were deliberately simplified and binary to minimize respondent fatigue, however this limits generalizability to real-world settings, where decision-making is often more nuanced. Clinicians must typically account for multilevel pathology, bilateral symptoms, history of prior epidural injections or spinal surgery, the presence of spinal hardware, anticoagulation status, and disease extending beyond a single spinal level, all of which were not reflected in the survey. Moreover, the ability of proceduralists to interpret their own imaging was not assessed as imaging findings were provided directly within the survey. Importantly, the survey did not require participants to justify their chosen approach, precluding deeper insight into the clinical reasoning behind each decision. Future studies should aim to incorporate more complex case scenarios (e.g., multilevel disease, multiple disc herniations) and solicit rationale for selected approaches to better understand the drivers behind clinical variability.

While most responses demonstrated strong consensus, a few questions revealed minor but clinically insignificant variability. These areas may benefit from further clarification and justification in future iterations of this research, particularly to address the underlying rationale guiding interventional choices.

## 5 Conclusion

This study aimed to identify the key factors shaping clinical decision-making among spinal interventionalists when selecting lumbar epidural steroid injection approaches based on survey responses from IPSIS members. The observed trends provide important insights to support the refinement of evidence-based treatment protocols and enhance the education of residents and fellows in interventional spine care.

Symptom pattern, particularly the presence of radicular pain, and nerve root correlation consistently influenced selection of the transforaminal supraneural approach regardless of MRI findings. This emphasizes the clinician's reliance on functional symptoms over structural imaging in guiding intervention.

Clinicians with PM&R backgrounds, formal pain fellowships, and recent training (<5 years) demonstrated greater concordance with evidence-based guidelines, including the use of non-particulate steroids and avoidance of use of particulate in L-TFESIs (as seen in Table 7). This suggests that education standardization is a powerful determinant of safe and consistent practice. Conversely, practitioners with more experience or from non-academic settings may rely more on personal experience or legacy habits, occasionally deviating from newer safety recommendations, highlighting an opportunity for ongoing continuing education in interventional spine care. The selection of dexamethasone for both interlaminar and transforaminal injections supports the growing shift toward safety-focused pharmacologic protocols, as emphasized in recent society guidelines and FDA safety alerts.

**Table 7:** Characteristics of respondent for most commonly selected injectate for TFESI.

		Frequency	Percentage
Most commonly selected medication	Dexamethasone	140	71.07 %
Residency Completed	Physical Medicine and Rehabilitation Residency	92	66.19 %
	Anesthesia Residency	36	25.90 %
Fellowship Completed	ACGME Pain Fellowship	50	35.71 %
	Non-accredited Spine Fellowship	22	15.71 %
	None	22	15.71 %
	NASS ISMM (Interventional Spine) Fellowship	19	13.57 %
Emphasis on IPSIS Guidelines in Training	Yes	99	70.71 %
	No	37	26.43 %
Years in Practice	Greater than 15 years	52	37.41 %
	Less than 5 years	44	31.65 %
	5–10 years	23	16.55 %
	10–15 years	20	14.39 %

Overall, the data suggest that training background and practice context are more predictive of guideline adherence than the clinical scenario itself, pointing to the need for ongoing reinforcement of standards across all practice environments.

We would like to thank Matthew Sherman for statistical assistance with this manuscript.

## Declaration of competing interest

The authors declare that they have no known competing financial interests or personal relationships that could have appeared to influence the work reported in this paper.
